# The Clinical Efficacy of Phytochemical Medicines Containing Tanshinol and Ligustrazine in the Treatment of Stable Angina: A Systematic Review and Meta-Analysis

**DOI:** 10.1155/2021/8616413

**Published:** 2021-02-02

**Authors:** Li Gao, Tong Wu, Juan Wang, Zhuoran Xiao, Chunhua Jia, Wei Wang

**Affiliations:** ^1^School of Traditional Chinese Medicine, Beijing University of Chinese Medicine, Beijing, China; ^2^St Michael's Hospital, University of Toronto, Toronto, M5B 1W8, Canada

## Abstract

**Background:**

Phytochemical medicines containing tanshinol and ligustrazine are commonly used in the treatment of stable angina in China, but their clinical effectiveness and risk have not been adequately assessed. In this paper, we conducted a systematic review and meta-analysis to evaluate the clinical efficacy.

**Methods:**

Relevant randomized controlled trials (RCTs) of phytochemical medicines containing tanshinol and ligustrazine in the treatment of stable angina were searched in electronic databases. The search date was up to March 31, 2020, and the languages of the RCTs were limited to English and Chinese.

**Results:**

A total of 28 studies, including 2518 patients, were included in the meta-analysis. It was shown that the adjunctive therapy of phytochemical medicines containing tanshinol and ligustrazine was better than the conventional therapies in the improvement of stable angina according to the clinical efficacy in symptoms (*n* = 2518, RR = 1.24, 95% CI: 1.20 to 1.29, *P* < 0.01) and clinical efficacy in electrocardiography (*n* = 1766, RR = 1.29, 95% CI: 1.19 to 1.40, *P* < 0.01).

**Conclusion:**

The meta-analysis supported the use of phytochemical medicines containing tanshinol and ligustrazine in the treatment of stable angina. However, quality of the evidence for this finding was low due to a high risk of bias in the included studies. Therefore, well-designed RCTs are still needed to further evaluate the efficacy.

## 1. Introduction

Stable angina is caused by fixed blockages in coronary arteries [1]. It typically occurs during activities, and the main symptoms are chest tightness and shortness of breath, which can be alleviated after a rest or administration of sublingual nitroglycerin [2–4]. Stable angina is a chronic coronary disease compared with unstable angina; however, it seriously affects patients' lives, such as restricting daily activities [5]. Then, the treatment aims to reduce morbidity and improve symptoms.

Currently, the main treatment of stable angina is medicine, such as nitroglycerin, beta-blockers, or calcium channel blockers, which focus on decreasing heart's workload and prevent episodes [6–9]. In China, phytochemical medicines are also used by many physicians. For example, Shao et al. [10] conducted a meta-analysis to assess the efficacy of danshen injection (main component: salvianic aid A) in the treatment of angina pectoris and concluded that it is more effective than antianginal agents alone. Yu et al. [11] and Wang et al. [12] conducted randomized controlled trials in the treatment of stable angina, respectively, and found that xinxuekang capsule (main component: steroidal saponins) had a better efficacy compared with danshen tablets. In addition, for the treatment of unstable angina, many researchers have supported different phytochemical medicines, such as puerarin injection [13], safflower yellow injection [14], and danshen chuanxiongqin injection [15].

In these phytochemical medicines, tanshinol and ligustrazine are the commonly used components. Tanshinol is also named salvianic aid A, with a molecular formula C_9_H_10_O_5_ [16]. Ligustrazine's molecular formula is C_8_H_12_N_2_ [17]. Tanshinol has antioxidant capacity [18]; it can attenuate oxidative stress by decreasing the expressions of FoxO3a signaling [19] and improve cardiovascular injury by scavenging reactive oxygen species [20]. In addition, tanshinol can attenuate endothelial cell apoptosis, which helps reduce the aortic atherosclerotic lesion area [21]. Ligustrazine has effects on calcium channels; the research of Ren et al. [22] showed that ligustrazine could significantly suppress calcium transient and contraction in rabbits. It was also reported that the ligustrazine exhibits an anti-inflammatory effect; as Guo et al. described, the salvia ligustrazine injection could decrease high-sensitivity C-reactive protein and interleukin-6 levels [17, 23]. Ligustrazine was also found to suppress acid-sensing ion channels and reduce ischemia-induced infarct size in rats with angina [24]. The combination of tanshinol and ligustrazine has efficacy in dilating coronary arteries, reducing blood viscosity, promoting blood circulation, and removing blood stasis through synergistic action [25–27]. Ye et al. [28] investigated the anti-inflammatory effect of danshen, chuanxiong, and their combination and found that their combination has a dual anti-inflammatory effect on macrophages and endothelial cells. All these findings provide a biological basis of tanshinol and ligustrazine in the treatment of angina.

Tanshinol and ligustrazine are the main compounds of danshen and chuanxiong. There are several phytochemical medicines whose main components are tanshinol and ligustrazine, such as danshen chuanxiongqin injection, guanxinning injection, and shenxiong glucose injection. Several systematic reviews have been conducted to evaluate the efficacy of these medicines in the treatment of angina pectoris. Jia et al. [29] analyzed eligible RCTs using guanxinning injection, Zhang et al. [15] assessed danshen chuanxiongqin injection in treating unstable angina pectoris, and Liu and Ding [30] assessed shenxiong glucose injection in the treatment of unstable angina pectoris. In addition, many randomized controlled trials have been published to support the use of danshen and chuanxiongqin in the treatment of stable angina. However, in the treatment of stable angina, no relevant meta-analysis has been conducted to assess the clinical efficacy or the risk of phytochemical medicines containing tanshinol and ligustrazine. Therefore, in this study, a meta-analysis was conducted to evaluate the efficacy of phytochemical medicines in the treatment of stable angina.

## 2. Methods

The protocol of this study was registered in PROSPERO with the registration number CRD42018105921.

### 2.1. Database and Search Strategies

The following electronic databases were searched by two independent reviewers (Gao L. and Wang J.): Web of Science, Cochrane Library, PubMed, Chinese Biomedical Literature Database, Chinese National Knowledge Infrastructure, Chinese Scientific Journal Database, and Wanfang Database. The search date was up to March 31, 2020, and the languages of the publications were limited to English and Chinese. The following search terms were used: (tanshinol OR salvianic acid A OR *β*-(3,4-dihydroxyphenyl) lactic acid OR danshensu OR danshen OR radix salvia OR salvia miltiorrhiza) AND (ligustrazine OR chuanxiong OR chuanxiongzine OR tetramethylpyrazine) AND (stable angina OR angina OR angina pectoris OR stenocardia OR angor pectoris) AND (randomized controlled trial).

### 2.2. Inclusion Criteria

The included studies must be RCTs.

  Participants: patients who were diagnosed with stable angina were included. The stable angina was diagnosed according to the criteria [31, 32], with tests such as electrocardiography (ECG), exercise ECG, and symptoms of the patients.
  Interventions: interventions using phytochemical medicines containing tanshinol and ligustrazine as a main treatment were chosen. The dosages of tanshinol and ligustrazine should be described specifically.
  Comparators: the control groups received conventional treatments, such as taking medicines to treat and prevent angina attacks. Placeboes were also included.
  Outcomes: the primary outcome was the clinical efficacy in symptoms and ECG; the secondary outcome is adverse event.

### 2.3. Exclusion Criteria

The exclusion criteria in the meta-analysis included (a) non-RCTs, case studies, experience summaries, animal experiments, and unpublished or repeated studies; (b) studies that used herbal medicines as the main intervention in addition to tanshinol and ligustrazine; (c) studies that used acupuncture or cupping as combined therapies; (d) patients who were identified as unstable angina; and (e) patients who have complications of heart failure, diabetes, stroke, or some other serious organic diseases.

### 2.4. Data Extraction and Quality Assessment

Four reviewers (Gao L, Wu T, Jia C, and Xiao Z) independently performed the data extraction and quality assessments. Meta-analysis was conducted using RevMan 5.3 software, and the risk of bias was assessed according to the Cochrane handbook [33]. Any disagreement was resolved by discussions among all reviewers.

## 3. Results

### 3.1. Description of the Included Studies

**Figure 1:**
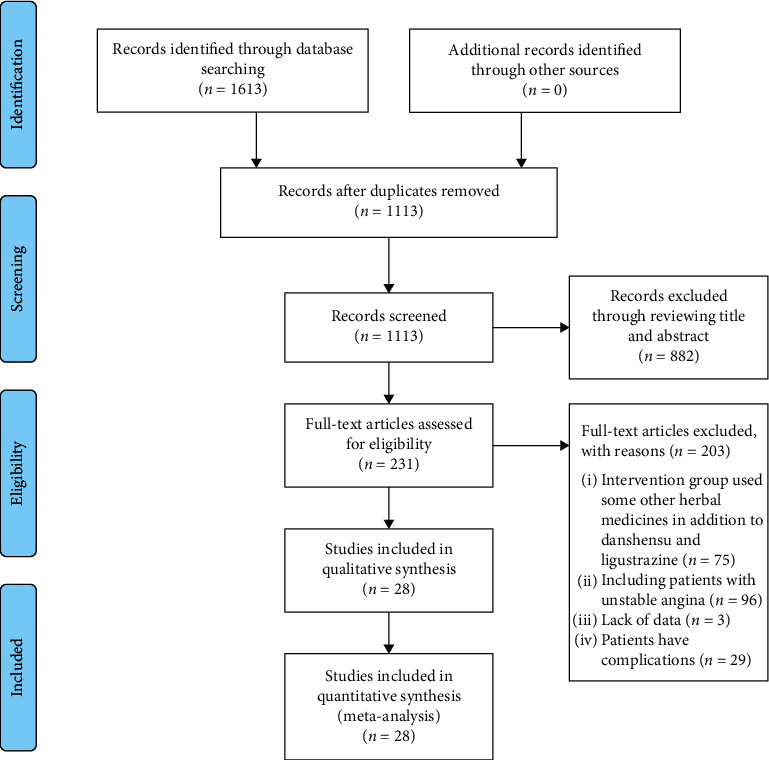
PRISMA flow diagram of the screening process.

In this meta-analysis, 1613 studies were identified through database searching. But 500 repeated studies were excluded and 882 irrelevant studies were excluded through title and abstract reviewing. The full texts of 231 studies were assessed and 203 studies were excluded, including 75 studies that used some other herbal medicines in addition to tanshinol and ligustrazine in the intervention group, 96 studies included patients with unstable angina, 2 study lacked data on the dosages of tanshinol and ligustrazine, 1 study lacked data to judge the efficacy, and 29 studies had patients with complications. At last, a total of 28 studies [34–61] were included in the meta-analysis. The screening process is summarized in a PRISMA flow diagram (Figure 1).

Details of the 28 studies are summarized in Table 1. There were 2518 patients in total, including 1276 patients in the intervention group and 1242 patients in the control group. Sample sizes of the studies were small, and only 8 studies had sample sizes greater than 100 patients. The youngest patient in these studies was 46 years old, while most of the studies reported patients older than 60 years old. Many patients had a long course of the disease, and the longest course was 25 years. In the control group, conventional treatments were used, such as nitroglycerin, beta-blockers, and calcium channel blockers. No study used a placebo. In the intervention group, phytochemical medicines containing tanshinol and ligustrazine were used based on the control group, except that one study that used a phytochemical medicine containing tanshinol and ligustrazine alone. The uses of tanshinol and ligustrazine were in different forms, 21 studies used danshen chuanxiongqin injection (DCI), 5 studies used shenxiong glucose injection (SGI), and 2 studies used danshen injection (DI) combined with ligustrazine injection (LI). The nature of constituents is botanical. 1 ml DCI contains 0.4 mg tanshinol and 20 mg ligustrazine, 1 ml SGI contains 0.2 mg tanshinol and 1 mg ligustrazine, and 1 ml DI contains 0.2 mg tanshinol. Details of the constituents of the 28 included studies are shown in Appendix table (available here). The treatment duration lasted from 7 days to 30 days. All the studies used clinical efficacy in symptoms as the main outcome, and 18 studies used clinical efficacy in ECG.

### 3.2. Risk of Bias

**Figure 2:**
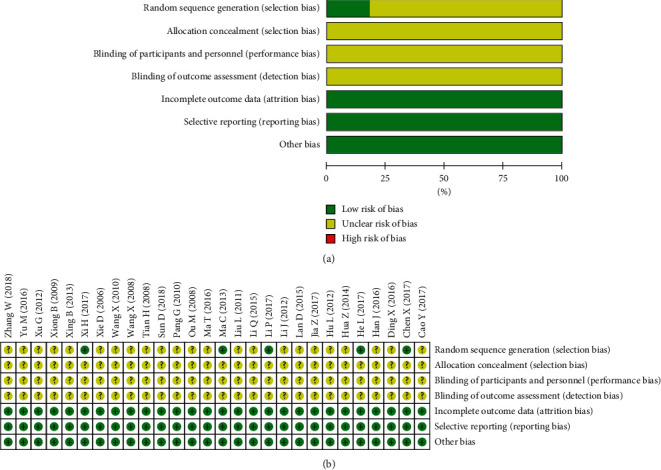
Risk of bias graph: (a) risk of bias in all included studies; (b) risk of bias summary.

The risk of bias was high in the included studies (Figure 2). All the studies were described using randomization, but only five of these studies [35, 38, 44, 47, 55] reported using an appropriate method of random sequence generation. None of the studies described the method for allocation concealment, blinding of participants and personnel, and blinding of the outcome assessment.

### 3.3. Outcome Measurements

The outcome measurements of the included studies include clinical efficacy in symptoms, clinical efficacy in ECG, and adverse events.

#### 3.3.1. Clinical Efficacy in Symptoms

The criteria for clinical efficacy in symptoms are defined as follows [62]: effective (the frequency of angina or the amount of nitroglycerine used is reduced by more than 50%) and no effect (the frequency of angina or the amount of nitroglycerine used is reduced by less than 50%).

**Figure 3:**
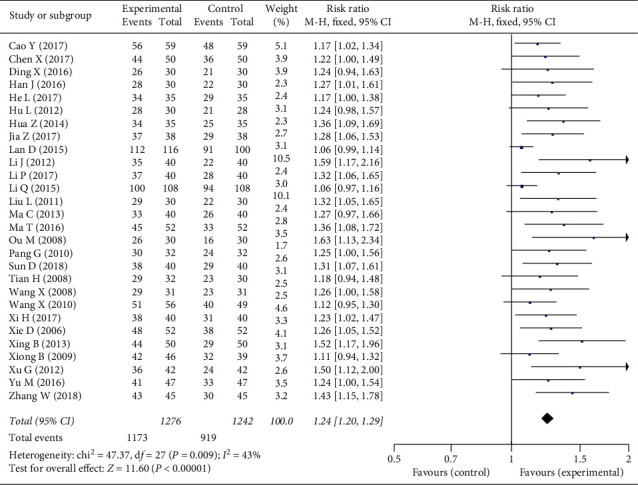
Forest plot of the clinical efficacy in symptoms of phytochemical medicines containing tanshinol and ligustrazine in the treatment of stable angina.

All the studies showed that phytochemical medicines containing tanshinol and ligustrazine have better clinical efficacy in symptoms. Since low heterogeneity was observed in the meta-analysis (*I*^2^ = 43%, which is lower than 50%), a model of fixed effects was used to calculate the pooled estimation with an analysis of the dichotomous data using relative risk (RR), including 95% confidence intervals (CIs). The total meta-analysis showed favorable effects of phytochemical medicines on clinical efficacy (*n* = 2518, RR = 1.24, 95% CI: 1.20 to 1.29, *P* < 0.01) compared with the control group (Figure 3).

#### 3.3.2. Clinical Efficacy in ECG

The criteria for clinical efficacy in ECG are defined as follows [62]: effective (recovery of ST-segment depression is more than 0.05 mV, or amplitude of the inverted T wave reduces more than 50%, or the shape of T wave changes from flat to upright) and no effect (no improvements in ECG compared with before).

**Figure 4:**
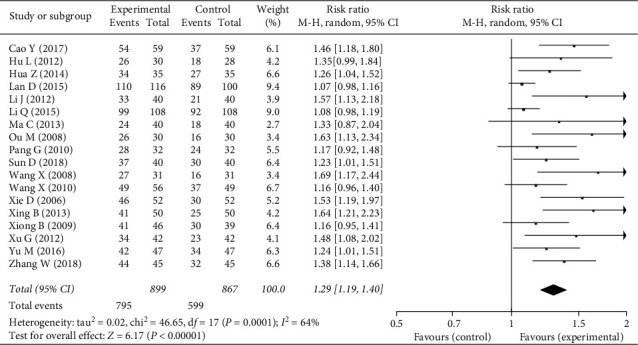
Forest plot of the clinical efficacy in ECG of phytochemical medicines containing tanshinol and ligustrazine in the treatment of stable angina.

Since high heterogeneity was observed in the meta-analysis (*I*^2^ = 64%, which is higher than 50%), a model of random effects was used. The total meta-analysis showed favorable effects of phytochemical medicines on ECG (*n* = 1766, RR = 1.29, 95% CI: 1.19 to 1.40, *P* < 0.01) compared with the control group (Figure 4).

#### 3.3.3. Adverse Events (AEs)

Only 10 studies reported AEs, of which 7 studies [35, 39, 40, 49, 54, 56, 59] reported that there were no AEs. In the other three studies [38, 42, 55], two studies reported AEs in the intervention group, including 2 cases of skin rash, 1 case of epigastric discomfort, 1 case of insomnia, and 1 case of tiredness, and three studies reported AEs in the control group, including 2 cases of nausea, 1 case of stomachache, 1 case of dizziness, 2 cases of skin rash, 4 cases of epigastric discomfort, 3 cases of insomnia, and 3 cases of tiredness. Other studies did not report AEs.

## 4. Discussion

Currently, phytochemical medicines containing tanshinol and ligustrazine have been widely utilized by physicians to treat stable angina in China. However, it is controversial since there was no systematic review to assess the therapy's clinical efficacy. Therefore, this meta-analysis aimed to evaluate the efficacy or risk of phytochemical medicines in the treatment of stable angina.

In this meta-analysis, DCI and SGI were used by most studies. Both DCI and SGI consist of tanshinol and ligustrazine. As reported, DCI and SGI have been studied in the treatment of acute myocardial infarction [63], myocardial ischemia/reperfusion injury [64], and focal cerebral ischemia [65]. In the theory of traditional Chinese medicine, angina pectoris should be treated by supplementing qi and activating blood circulation. Tanshinol and ligustrazine are extracted from danshen and chuanxiong, which are two commonly used herbs in the treatment of cardiac diseases in China.

Tanshinol is the drug used for promoting blood circulation and removing blood stasis, which can improve cardiac function by increasing coronary blood flow and slowing the heart rate down [66]. Clinical practice has proved that salvia has a curative effect on myocardial hypoxia caused by myocardial infarction, and it plays a role in anticoagulation by dilating peripheral vessels to reduce blood pressure and improving cAMP (cyclic adenosine monophosphate) in cells [67, 68]. Ligustrazine is a kind of active alkaloid, which is effective to dilate coronary arteries and reduce coronary resistance. Ligustrazine is efficient in increasing coronary blood flow and improving myocardial oxygen supply, making it commonly to be used to inhibit platelet aggregation and depolymerize the aggregated platelets [69, 70]. In the treatment of cardiac diseases, tanshinol and ligustrazine can promote blood circulation, dilate coronary arteries, and inhibit platelet aggregation [29, 71], which provides a rationale in the treatment of stable angina.

Heterogeneity in this meta-analysis was moderate, with clinical efficacy in symptoms of *I*^2^ = 43%, and clinical efficacy in ECG of *I*^2^ = 64%. The reasons for this may be that different conventional treatments were used in different control groups. Therapies in the intervention group were based on the control group; different therapies make the efficacy hard to be assessed.

The high risk of bias of the included studies makes the methodological quality for this finding low. There are several limitations to this systematic review. First, for most of the included studies, the methods for randomization, allocation concealment, and blinding were not reported clearly. Second, in the 28 included studies, only 8 studies had sample sizes greater than 100 patients, and the small sample sizes in most studies made meaningful conclusions difficult to be drawn. Third, clinical efficacy was the main outcome measurement for most studies, but a bias from the physicians may decrease the reliability and validity of the studies. Fourth, all the studies were published in China, which may limit the generalization of the findings.

## 5. Conclusion

In conclusion, this meta-analysis included 28 studies that used phytochemical medicines containing tanshinol and ligustrazine in the treatment of stable angina, and the results supported their clinical application. However, the studies analyzed to date are of relatively low quality. More rigorous RCTs with large sample sizes are needed to further evaluate the clinical efficacy and the adverse effects.

## Figures and Tables

**Table 1:** Details of the 28 included studies on phytochemical medicines containing tanshinol and ligustrazine in the treatment of stable angina.

Study	Sample size	Age (years)	Course of disease (years)	Intervention group	Control group	Treatment duration (days)	Main outcomes
Cao and Wang [34]	118 (59/59)	63.0 ± 14.0 61.5 ± 14.5	NR	DCI (10 ml) + TCR	Nifedipine 30–60 mg/d; metoprolol 100–200 mg/d; aspirin 100–300 mg/d; nitroglycerin when necessary	14	CES + ECG
Chen [35]	100 (50/50)	57.24 ± 9.64	NR	DCI (10 ml)	Aspirin 100 mg/d; atorvastatin 20 mg/d; trimetazidine 60 mg/d; nitroglycerin 10 mg/d	7	CES
Ding [36]	60 (30/30)	56–72	NR	DCI (10 ml) + TCR	Nitroglycerin	10	CES
Han [37]	60 (30/30)	64.9 ± 5.89 65.7 ± 7.93	NR	DCI (10 ml) + TCR	Nitrates; aspirin; calcium channel blockers	14	CES
He and Li [38]	70 (35/35)	48.2 ± 2.1	NR	DCI (10 ml) + TCR	Aspirin; nitrates	14	CES
Hu [39]	58 (30/28)	60 ± 8 59 ± 9	NR	DCI (10 ml) + TCR	Nitrates; beta-blockers; calcium channel blockers; aspirin	14	CES + ECG
Hua et al. [40]	70 (35/35)	64.7 ± 8.4 63.3 ± 8.3	0.5–10	DCI (10 ml) + TCR	Isosorbide mononitrate 25 mg/d; quinapril 10 mg/d; metoprolol 50 mg/d; aspirin 100 mg/d	14	CES + ECG
Jia [41]	76 (38/38)	62.04 ± 2.15 61.92 ± 2.13	NR	DCI (10 ml) + TCR	Aspirin 100 mg/d; atorvastatin 20 mg/d; trimetazidine 60 mg/d; isosorbide mononitrate 40 mg/d	7	CES
Lan [42]	216 (116/100)	57.6 ± 4.6 58.1 ± 5.2	7.62 ± 3.87 8.91 ± 4.28	DCI (10 ml) + TCR	Nitrates; beta-blockers; calcium channel blockers; aspirin	14	CES + ECG
Li et al. [43]	80 (40/40)	67.3 ± 6.20	3.5 ± 1.6	DCI (10 ml) + TCR	Isosorbide mononitrate 40 mg/d; metoprolol 47.5 mg/d; aspirin 100 mg/d; trimetazidine 60 mg/d	14	CES + ECG
Li and Li [44]	80 (40/40)	58.93 ± 2.07 58.42 ± 2.31	4.45 ± 1.43 4.37 ± 1.52	DCI (10 ml) + TCR	Aspirin 100 mg/d; atorvastatin 20 mg/d; trimetazidine 60 mg/d; isosorbide mononitrate 40 mg/d	7	CES
Li [45]	216 (108/108)	64.7 ± 4.8	10.6 ± 1.2	DCI (10 ml) + TCR	Aspirin; calcium channel blockers; beta-blockers; nitrates	14	CES + ECG
Liu and Li [46]	60 (30/30)	63.7 ± 7.7 64.9 ± 9.4	NR	SGI (100 ml) + TCR	Nitrates; aspirin; beta-blockers; calcium channel blockers; ACE inhibitors; ARBs	14	CES
Ma et al. [47]	80 (40/40)	NR	NR	DCI (10 ml) + TCR	Nitrates; beta-blockers; calcium channel blockers; aspirin	14	CES + ECG
Ma et al. [48]	104 (52/52)	63.74 ± 11.83 62.34 ± 10.63	NR	SGI (100 ml) + TCR	Aspirin 100 mg/d; isosorbide mononitrate 40 mg/d; metoprolol 50 mg/d; atorvastatin 20 mg/d	14	CES
Ou [49]	60 (30/30)	60.33 ± 10.04 61.21 ± 9.36	0.08–12 0.16–13	SGI (100 ml) + TCR	Nitrates; beta-blockers; calcium channel blockers; antiplatelet drug	14	CES + ECG
Pang and Liu [50]	64 (32/32)	77.2 ± 5.6	6–20	SGI (200 ml) + TCR	Antiplatelet drug; beta-blockers; statins; ACE inhibitors; ARBs; nitrates; lipid-lowering drug; hypotensor; nitroglycerin; isosorbide mononitrate 20 mg/d	14	CES + ECG
Sun [51]	80 (40/40)	72.3 ± 0.2 71.9 ± 0.4	5.2 ± 0.6 5.1 ± 0.4	DCI (5 ml) + TCR	Antiplatelet drug; thrombolytic drug; lipid-lowering drug; hypotensor; digoxin when necessary	30	CES + ECG
Tian [52]	62 (32/30)	61.68 ± 10.98 60.39 ± 9.76	NR	DCI (20 ml) + TCR	Nitrates; calcium channel blockers	14	CES
Wang and Wang [54]	62 (31/31)	46–58	3–18	DI (20 ml) + LI (80 mg) + TCR	Nitrates; beta-blockers; calcium channel blockers	14	CES + ECG
Wang and Lian [53]	105 (56/49)	51–75	1.6–25	DCI (10 ml) + TCR	Nitrates; beta-blockers; calcium channel blockers; aspirin	14	CES + ECG
Xi [55]	80 (40/40)	59.3 ± 6.4 62.4 ± 5.3	5.9 ± 0.6 4.8 ± 0.8	DCI (10 ml) + TCR	Aspirin; atorvastatin; nitroglycerin	14	CES
Xie and Zhu [56]	104 (52/52)	51–79	1.6–25	DI (20–30 ml) + LI (40–80 mg) + TCR	Nitrates; beta-blockers; calcium channel blockers; aspirin	14	CES + ECG
Xing [57]	100 (50/50)	66.2 ± 5.60	2.8 ± 1.8	DCI (10 ml) + TCR	Isosorbide mononitrate 40 mg/d; aspirin 100 mg/d	14	CES + ECG
Xiong and Wang [58]	85 (46/39)	52–71	1.6–25	DCI (10 ml) + TCR	Nitrates; beta-blockers; calcium channel blockers; aspirin	14	CES + ECG
Xu and Qin [59]	84 (42/42)	56.14 ± 7.40 51.20 ± 7.30	1–13 2–12	DCI (10 ml) + TCR	Nitrates; aspirin; beta-blockers; nitroglycerin when necessary	14	CES + ECG
Yu and Fang [60]	94 (47/47)	NR	NR	DCI (5–10 ml) + TCR	Antiplatelet drug; thrombolytic drug; lipid-lowering drug; hypotensor; digoxin when necessary	20	CES + ECG
Zhang [61]	90 (45/45)	68.21 ± 9.26 68.52 ± 9.39	NR	SGI (200 ml) + TCR	Antiplatelet drug; anticoagulant; ACE inhibitors; beta-blockers; calcium channel blockers; statins; nitrates	10–14	CES + ECG

NR: not reported; DCI: danshen chuanxiongqin injection; SGI: shenxiong glucose injection; DI: danshen injection; LI: ligustrazine injection; GI: guanxinning injection; TCR: treatments in the control group; ACE: angiotensin-converting enzyme; ARBs: angiotensin receptor blockers; CES: clinical efficacy in symptoms; ECG: electrocardiography.

## Data Availability

All data generated or analyzed during this study are included in this published article and its supplementary information files.

## Abbreviations

RCTs: Randomized controlled trials
ECG: Electrocardiography
DCI: Danshen chuanxiongqin injection
SGI: Shenxiong glucose injection
DI: Danshen injection
LI: Ligustrazine injection
CIs: Confidence intervals
AEs: Adverse events
TCR: Treatments in the control group
ACE: Angiotensin-converting enzyme
ARBs: Angiotensin receptor blockers
CES: Clinical efficacy in symptoms.

## Additional Points

*Highlights.* (i) It was the first meta-analysis assessing the efficacy of phytochemical medicines containing tanshinol and ligustrazine in the treatment of stable angina. (ii) The clinical efficacy and safety of phytochemical medicines containing tanshinol and ligustrazine in the treatment of stable angina were comprehensively assessed. (iii) The efficacy of phytochemical medicines containing tanshinol and ligustrazine in the treatment of stable angina needed to be further evaluated.

## Supplementary Materials

Supplementary Materials File name: Appendix table. Title of data: summary table of the constituents of the 28 included studies. Description of data: statements of the constituents of the included studies.
