# Supplementary material for: The Clinical Efficacy of Phytochemical Medicines Containing Tanshinol and Ligustrazine in the Treatment of Stable Angina: A Systematic Review and Meta-Analysis
Source: Evid Based Complement Alternat Med. 2021 Feb 2;2021:8616413. doi: 10.1155/2021/8616413 (PMC7875616; doi:10.1155/2021/8616413)
Supplement: Supplementary Materials — File name: Appendix table. Title of data: summary table of the constituents of the 28 included studies. Description of data: statements of the constituents of the included studies. [file 8616413.f1.docx]

Summary table of the constituents of the 28 included studies

| Study | Intervention group | Statements of the constituents |
| --- | --- | --- |
| Cao Y (2017) | DCI(10ml) | 1 ml DCI contains 0.4 mg tanshinol and 20 mg ligustrazine. The DCI was bought from Guizhou Baite Pharmaceutical Co., Ltd. The use of DCI is to add 10 ml of DCI to 500 ml of normal saline or 5% glucose injection. |
| Chen X (2017) | DCI(10ml) | 1 ml DCI contains 0.4 mg tanshinol and 20 mg ligustrazine. The source of DIC was not reported. The use of DCI is to add 10 ml of DCI to 250 ml of normal saline injection. |
| Ding X (2016) | DCI(10ml) | 1 ml DCI contains 0.4 mg tanshinol and 20 mg ligustrazine. The source of DIC was not reported. The use of DCI is to add 10 ml of DCI to 5% glucose injection. |
| Han J (2016) | DCI(10ml) | 1 ml DCI contains 0.4 mg tanshinol and 20 mg ligustrazine. The source of DIC was not reported. The use of DCI is to add 10 ml of DCI to 500 ml of normal saline or 5% glucose injection. |
| He L (2017) | DCI(10ml) | 1 ml DCI contains 0.4 mg tanshinol and 20 mg ligustrazine. The source of DIC was not reported. The use of DCI is to add 10 ml of DCI to 250 ml of normal saline injection. |
| Hu L (2012) | DCI(10ml) | 1 ml DCI contains 0.4 mg tanshinol and 20 mg ligustrazine. The source of DIC was not reported. The use of DCI is to add 10 ml of DCI to 250 ml of normal saline or 5% glucose injection. |
| Hua Z (2014) | DCI(10ml) | 1 ml DCI contains 0.4 mg tanshinol and 20 mg ligustrazine. The source of DIC was not reported. The use of DCI is to add 10 ml of DCI to 250 ml of 5% glucose injection. |
| Jia Z (2017) | DCI(10ml) | 1 ml DCI contains 0.4 mg tanshinol and 20 mg ligustrazine. The DCI was bought from Guizhou Baite Pharmaceutical Co., Ltd. The use of DCI is to add 10 ml of DCI to 250 ml of normal saline injection. |
| Lan D (2015) | DCI(10ml) | 1 ml DCI contains 0.4 mg tanshinol and 20 mg ligustrazine. The DCI was bought from Guizhou Baite Pharmaceutical Co., Ltd. The use of DCI is to add 10 ml of DCI to 500 ml of normal saline or 5% glucose injection. |
| Li J (2012) | DCI(10ml) | 1 ml DCI contains 0.4 mg tanshinol and 20 mg ligustrazine. The DCI was bought from Guizhou Baite Pharmaceutical Co., Ltd. The use of DCI is to add 10 ml of DCI to 250 ml of normal saline or 5% glucose injection. |
| Li P (2017) | DCI(10ml) | 1 ml DCI contains 0.4 mg tanshinol and 20 mg ligustrazine. The DCI was bought from Guizhou Baite Pharmaceutical Co., Ltd. The use of DCI is to add 10 ml of DCI to 250 ml of 5% glucose injection. |
| Li Q (2015) | DCI(10ml) | 1 ml DCI contains 0.4 mg tanshinol and 20 mg ligustrazine. The DCI was bought from Guizhou Baite Pharmaceutical Co., Ltd. The use of DCI is to add 10 ml of DCI to 250 ml of 5% glucose injection. |
| Liu L (2011) | SGI(100ml) | 1 ml SGI contains 0.2 mg tanshinol and 1 mg ligustrazine. The source of SGI was not reported. The SGI is used through intravenous injection. |
| Ma C (2013) | DCI(10ml) | 1 ml DCI contains 0.4 mg tanshinol and 20 mg ligustrazine. The source of DIC was not reported. The use of DCI is to add 10 ml of DCI to 250 ml of 5% glucose injection. |
| Ma T (2016) | SGI(100ml) | 1 ml SGI contains 0.2 mg tanshinol and 1 mg ligustrazine. The source of SGI was not reported. The SGI is used through intravenous injection. |
| Ou M (2008) | SGI(100ml) | 1 ml SGI contains 0.2 mg tanshinol and 1 mg ligustrazine. The SGI was bought from Guizhou Yibai Pharmaceutical Co., Ltd. The SGI is used through intravenous injection. |
| Pang G (2010) | SGI(200ml) | 1 ml SGI contains 0.2 mg tanshinol and 1 mg ligustrazine. The source of SGI was not reported. The SGI is used through intravenous injection. |
| Sun D (2018) | DCI(5ml) | 1 ml DCI contains 0.4 mg tanshinol and 20 mg ligustrazine. The source of DIC was not reported. The use of DCI is to add 5 ml of DCI to 250 ml of 5% glucose injection. |
| Tian H (2008) | DCI(20ml) | 1 ml DCI contains 0.4 mg tanshinol and 20 mg ligustrazine. The source of DIC was not reported. The use of DCI is to add 20 ml of DCI to 250 ml of 5% glucose injection. |
| Wang X (2008) | DI(20ml) +LI(80mg) | 1 ml DI contains 0.2 mg tanshinol. The sources of DI and LI were not reported. The use of DI is to add 20 ml of DI to 250 ml of 5% glucose injection, the use of LI is to add 80 mg of ligustrazine to 250 ml of 5% glucose injection. |
| Wang X (2010) | DCI(10ml) | 1 ml DCI contains 0.4 mg tanshinol and 20 mg ligustrazine. The source of DIC was not reported. The use of DCI is to add 10 ml of DCI to 250 ml of 5% glucose injection. |
| Xi H (2017) | DCI(10ml) | 1 ml DCI contains 0.4 mg tanshinol and 20 mg ligustrazine. The DCI was bought from Jilin Sichang Pharmaceutical Co., Ltd. The use of DCI is to add 10 ml of DCI to 250 ml of 5% glucose injection. |
| Xie D (2006) | DI(20-30ml)+LI(40-80mg) | 1 ml DI contains 0.2 mg tanshinol. The sources of DI and LI were not reported. The use of DI is to add 20-30 ml of DI to 250 ml of 5% glucose injection, the use of LI is to add 40-80 mg of ligustrazine to 250 ml of 5% glucose injection. |
| Xing B (2013) | DCI(10ml) | 1 ml DCI contains 0.4 mg tanshinol and 20 mg ligustrazine. The source of DIC was not reported. The use of DCI is to add 10 ml of DCI to 250 ml of normal saline injection. |
| Xiong B (2009) | DCI(10ml) | 1 ml DCI contains 0.4 mg tanshinol and 20 mg ligustrazine. The DCI was bought from Guizhou Baite Pharmaceutical Co., Ltd. The use of DCI is to add 10 ml of DCI to 250 ml of 5% glucose injection. |
| Xu G (2012) | DCI(10ml) | 1 ml DCI contains 0.4 mg tanshinol and 20 mg ligustrazine. The source of DIC was not reported. The use of DCI is to add 10 ml of DCI to 250 ml of 5% glucose injection. |
| Yu M (2016) | DCI(5-10ml) | 1 ml DCI contains 0.4 mg tanshinol and 20 mg ligustrazine. The DCI was bought from Guizhou Baite Pharmaceutical Co., Ltd. The use of DCI is to add 5-10 ml of DCI to 250-500 ml of 5% glucose injection. |
| Zhang W (2018) | SGI(200ml) | 1 ml SGI contains 0.2 mg tanshinol and 1 mg ligustrazine. The SGI was bought from Kunming Pharmaceutical Co., Ltd. The SGI is used through intravenous injection. |

DCI: danshen chuanxiongqin injection; SGI: shenxiong glucose injection; DI: danshen injection; LI: ligustrazine injection.
